# Progress in Cancer

**Published:** 1904-04-02

**Authors:** 


*(To be continued.)*

### Progress in Cancer.

**Dr. Otto Schmidt's Specific Treatment of Cancer.**—Jossè Johnson,[1 2 3] having spent some time in Cologne expressly to study Dr. Schmidt's method, read a paper on the subject before the Abernethian Society at St. Bartholomew's Hospital. He said that the method was based on the parasitic origin of cancer. This theory is that a living parasite enters the body and then finds an anaplastic abnormal cell. The parasite irritates this cell to division and to produce new cells of its own kind, and into these new cells the progeny of the parasite enters. The aggregations of cells form malignant growths, and wandering cells carrying the parasite form the metastatic deposits. Any treatment to be successful must be anti-parasitic and not anti-toxic, therefore by some means anti-bodies must be introduced into the blood of the patient, that is, he must be actively immunised; or else he must be passively immunised by injecting a serum containing anti-bodies. This is the rationale of Schmidt's method. In 1902 he succeeded in cultivating artificially a parasite which fulfilled most of the conditions necessary to prove its specificity. 1. It was found in every malignant growth examined. 2. It was capable of artificial cultivation in pure culture. 3. In two cases injections of pure culture produced tumours in white mice. 4. The same parasite was found in these tumours, and from them was again cultivated in pure culture. 5. The parasite does not occur in benign tumours. The rationale of treatment is twofold. Firstly, active immunisation—that is, by the injection of cultures 14 to 21 days old, which have been first killed by the application of heat (65° C.); and secondly, passive immunisation by the injection of the serum of immunised animals, sheep and horses, into the subcutaneous tissues of the patient. The treatment is commenced in a very mild form. On the first day 1-100th of a milligramme of the sterilised culture is given, on the second day 1-10th, on the third day 2-10ths, and on the fourth day 1 milligramme. These doses are usually sufficient to produce the characteristic reaction. The serum is used in doses of 20 centigrammes. The injection should not be made near the tumour itself. Injections of the cultures into a healthy individual produced no symptoms; but in the cancerous, eight or ten hours after the second or third injection there was a sense of malaise with more or less rise of temperature. (The cachectic patients whose blood is already poisoned with toxins often do not react at all.) At the same time there occur swelling of the tumour and of any metastatic deposits or infected glands, with pain and tenderness of all affected parts. The specific reaction is an inflammatory process. The activity of the reaction varies very much and diminishes as the patient becomes immune. The inflammatory process causes an emigration of leucocytes, which is necessary for the destruction of the parasite; the anti-bodies being able to only weaken the parasites. Retrogressive changes with the development of much fibrous tissue occur in the growths as the result of the injections. The relative value of active and passive immunisation is at present uncertain. The impression is that the injections of serum alone are enough to inhibit the growth of the primary tumour, and prevent the formation of metastatic deposits only temporarily. The serum improves the general state, but is open to the objections of all serums that it may cause erythema, pain in the back, etc. Dr. Johnson only spoke of the method as one of treatment, not of cure, and thought its most useful application would be as an immediate sequence to operation to destroy the remaining parasites and cells, and to immunise the patient from further attacks. Its value also for diagnostic purposes was referred to. A number of cases are reported in the paper as having been treated by this method, many with much improvement, and at any rate none were any worse, having been observed for five or six months. Mr. D'Arcy Power[4] records with great detail three cases that he treated by Schmidt's method, Dr. Johnson supplying the fluid. All three cases had been recently operated on and shown by microscopic examination to be cancerous. Mr. Power arrives at the following conclusions: 1. There is no doubt a reaction takes place after injection of Schmidt's serum. The temperature rises and with it the pulse, but the respirations are not affected. There is nothing peculiar in this rise of temperature for it follows the injection of serum which contains toxins and, in all probability, any similar active serum would produce a similar result. It cannot be said with accuracy that the reaction is specific, as the first case showed a most typical reaction, but subsequent examination of her tissues failed to detect any malignant disease. 2. The local effect upon the tumour was shown in each case: the breast in the first, and the malignant masses in the others, became inflamed and reddened. The serum did not, however, appear to act by selection on malignant tissues only, but appeared to intensify any pre-existing inflammation. In the first case, a cancer of the left breast, the glands in both axillæ became enlarged, though subsequent examination showed that none of the glands were affected with cancer. 3. The treatment is painful apart from the succession of hypodermic injections. 4. Malignant disease progresses, even to a fatal issue, while the injections are being given. Mr. Power concludes that, so far as his experience goes, the method is of no service from a prophylactic, diagnostic, or curative standpoint.

**On the Etiology of Carcinoma.**—Monsarrat,[5] since a previous report in 1899, has been continuing his researches into the etiology of carcinoma. These researches have been confined to one type of carcinoma, that affecting the female breast, and they have provided material out of which he has been able to construct the life history of the organism and have also afforded evidence of interest and importance relating to the question whether the organism is etiologically connected with the disease. The paper deals fully with the isolation of the organism, the morphology and life cycle of the organism, the appearances presented by the organism after inoculation into animals, and the lesions produced in animals by inoculation of the organism. The following summary of researches is given at the end of the paper: 1. From a considerable proportion (58.3 per cent.) of specimens of carcinoma mammæ an organism presenting characteristic features was isolated. 2. This organism presents a life-history in which two cycles were traced—the one a vegetative budding cycle, the other a sporulating cycle. 3. The organism when injected into animals is capable of infecting and inhabiting endothelial and epithelial cells. 4. The organism initiates in endothelium and epithelium a process of proliferation, as a result of which masses of new-formed tissue are built up, which consist of a parenchyma and a stroma, and grow and extend actively from their centres of origin. 5. This new cell-mass formation may be associated with growth of a similar character in neighbouring glands, and some evidence was also provided that visceral metastasis occurs. 6. Intracellular bodies are demonstrable in carcinomata mammæ, which present the same features as the intracellular parasites of the experimentally produced nodules. 7. The evidence derived from these researches points to the conclusion that the organism described is an etiological factor in the morbid process known as carcinoma mammæ.

[1] Lancet, Nov. 14, 1903. [2] Brit. Med. Jour., Nov. 14, 1903. [3] Med. Rec., Dec. 12, 1903. [4] Brit. Med. Jour., Feb. 6, 1904. [5] Ibid., Jan. 23, 1904.

